# DNA damage and cellular abnormalities in tuberculosis, lung cancer and chronic obstructive pulmonary disease

**DOI:** 10.1186/s40248-015-0034-z

**Published:** 2015-12-19

**Authors:** Andréa Lúcia Gonçalves da Silva, Maribel Josimara Bresciani, Thaís Evelyn Karnopp, Augusto Ferreira Weber, Joel Henrique Ellwanger, João Antonio Pêgas Henriques, Andréia Rosane de Moura Valim, Lia Gonçalves Possuelo

**Affiliations:** Department of Health and Physical Education, University of Santa Cruz do Sul – UNISC, Avenida Independência, 2293, Bairro Universitário, Santa Cruz do Sul, RS CEP 96815-900 Brazil; Department of Biology and Pharmacy, University of Santa Cruz do Sul - UNISC, Santa Cruz do Sul, RS Brazil; Graduate Program in Cellular and Molecular Biology, Federal University of Rio Grande do Sul - UFRGS, Porto Alegre, RS Brazil; Graduate Program in Health Promotion, University of Santa Cruz do Sul - UNISC, Santa Cruz do Sul, RS Brazil

**Keywords:** Apoptosis, Micronucleus, Necrosis, Pulmonary diseases

## Abstract

**Background:**

Tuberculosis (TB), Lung Cancer (LC) and Chronic Obstructive Pulmonary Diseases (COPD) affect millions of individuals worldwide. Monitoring of DNA damage in pathological situations has been investigated because it can add a new dimension to clinical expression and may represent a potential target for therapeutic intervention. The aim of this study was to evaluate DNA damage and the frequency of cellular abnormalities in TB, LC and COPD patients by comparing them to healthy subjects.

**Methods:**

The detection of DNA damage by a buccal micronucleus cytome assay was investigated in patients with COPD (*n* = 28), LC (*n* = 18) and TB (*n* = 22) and compared to control individuals (*n* = 17).

**Results:**

The COPD group had a higher frequency of apoptotic cells compared to TB and LC group. The TB group showed a higher frequency of DNA damage, defect in cytokinesis, apoptotic and necrotic cells. Patients with LC had low frequency of chromosomal aberrations than TB and COPD patients.

**Conclusion:**

COPD patients showed cellular abnormalities that corresponded to cell death by apoptosis and necrosis, while patients with TB presented defects in cytokinesis and dysfunctions in DNA repair that resulted in the formation of micronucleus (MN) besides apoptotic and necrotic cells. Patients with COPD, TB and LC had a low frequency of permanent DNA damage.

## Background

The prevalence of chronic obstructive pulmonary disease (COPD) in Brazil is estimated at 7.3 million individuals [1]. The most recent global estimate indicated an incidence of 1.82 million new cases of lung cancer (LC) in 2012 [2]. In 2013, there were approximately 9.0 million people with tuberculosis (TB) worldwide [3].

Constant oxidative stress in the lung cells of the elderly serves as a source of free radicals that damage the DNA of pulmonary and circulating cells [4–6], thereby contributing to the pathogenesis of lung diseases. However, little is known about the levels of DNA damage in the three diseases evaluated in this study.

The synergistic effects of breathable particles lead to oxidative stress. These particles have a high carcinogenic potential and can lead to the increased production of pulmonary inflammatory mediators that cause oxidative damage to all major cellular components (i.e., membrane lipids, proteins, and DNA) [4–6]. Genomic instability is characterized by an increased frequency of changes in the genetic material of the cells. Loss of genomic stability is one of the most important aspects of mutagenesis and the genetic changes associated with carcinogenesis. One way to detect genomic instability is to quantify the frequency of chromosomal alterations induced by mutagens. It is currently accepted that DNA damage directly or indirectly caused by mutagenic and carcinogenic agents is primarily responsible for chromosomal abnormalities. Therefore, DNA damage deserves special attention from health centers and the scientific community, and its monitoring is useful for routine testing to prevent different pathologies [7].

The micronucleus (MN) in dividing cells forms as a result of chromosome breakage due to unrepaired or misrepaired DNA lesions. Chromosomal malsegregation due to mitotic malfunction can also result in the formation of the MN [8]. These events may be induced by oxidative stress, exposure to clastogens, or genetic defects that affect the cell cycle and/or DNA repair genes. Deficiencies in the nutrients that function as required co-factors for DNA metabolism can also play a role in the process of MN formation [8–10]. All these molecular events are associated with the formation of the MN and they are observed in patients with lung diseases [4, 8, 11]. The MN assay in exfoliated cells can be used as an internal dosimeter of DNA damage in specific tissues. The oral epithelium is in constant contact with genotoxic environmental agents; therefore, it is an important target site of inhaled or ingested toxic substances [12]. Damage to the oral epithelium indirectly indicates damage to lung cells, because the damage-inducing agents that enter the body through the oral epithelium will also reach the lungs.

The risk of mortality in patients with COPD, LC or TB is directly related to the exacerbation and progression of each disease. Oxidants are formed due to an exacerbated inflammatory process, especially in individuals with an impaired immune response [6, 13, 14]. Genetic and environmental factors, DNA integrity and lifestyle respectively, together with clinical parameters, may contribute to personalize clinical process and therapeutic strategy [15]. Therefore, the aim of this study was to evaluate permanent DNA damage in patients with COPD, LC and TB by comparing them to healthy subjects, and to identify the frequency of cellular abnormalities in these individuals.

## Methods

A case-control study was performed with 28 patients with COPD (age 64.21 ± 8.20), 18 with LC (age 65.06 ± 6.64), and 22 with TB (age 36.09 ± 16.25) at the beginning of medical treatment. The control group was composed of healthy subjects with preserved lung functions (*n* = 17, age 62.82 ± 4.78). Data collection and patient sampling were performed in the following locations: COPD - Cardio Respiratory Rehabilitation Program at Santa Cruz Hospital (SCH, Rio Grande do Sul, Brazil); LC - Integrated Oncology Center at Ana Nery Hospital (Rio Grande do Sul, Brazil); TB - TB Clinic at SCH.

Individuals with preserved lung functions that were exposed to the same living conditions and were similar in terms of gender, age and body mass index (BMI) were included in the control group. These individuals were recruited from the Family Health Units located at Rio Pardo and Taquari Valleys (Rio Grande do Sul, Brazil). The protocol of this study was approved by the Ethics Committee of the University of Santa Cruz do Sul - UNISC (protocol number: 374.298). All participants answered a personal health questionnaire and signed an informed consent form.

### Buccal micronucleus cytome assay (BMCyt)

We adapted the BMCyt assay from the method described by Thomas et al. [16]. Buccal cell samples were collected and processed in accordance with the same authors. The cells were directly fixed in methanol (an adaptation of the original protocol). For each subject, two microtubes were prepared with methanol for left cheek and right cheek cells. The cells were collected by rotating a cytobrush in a spiral motion 20 times against the inner surface of the cheek wall. The head of the cytobrush was placed into its respective micro tube containing 1000 μL of methanol [17, 18]. Then, the samples were transported to the laboratory and kept under refrigeration (5 °C) prior to performing the micronucleus test.

The cells were centrifuged and washed with methanol after the supernatant has been removed to concentrate a larger number of cells. Approximately 200 μ L of the cell suspension was spread over a microscope slide and allowed to dry. Then, the slides were placed in a staining jar containing 50 % ethanol for 1 min and immediately transferred to a staining jar containing 20 % ethanol for 1 additional minute. After washing the slides with deionized water for 2 min, hydrochloric acid 5 M was added for 30 min to allow cell hydrolysis.

After cell hydrolysis, the slides were washed in tap water for 5 min and then with distilled water for 1 min. Subsequently, the slides were maintained for 1 h and 20 min in Schiff's reagent for staining and then washed with distilled water. After staining, the cells were counterstained with Fast Green for 20 seconds and then washed with distilled water for 2 min. The slides/cells were dried at room temperature and stored for microscopic analysis. Slide analysis was performed single-blind using a conventional optical microscope at a 400X magnification. A total of 2,000 cells per slide were analyzed, for a total count of 4,000 cells per sample.

### Cell classification

The BMCyt can be used as an indicator of DNA damage (MN and/or nuclear buds - BUDS), cytokinesis defects (binucleated cells - BC), proliferation potential (basal cell frequency), apoptotic cell death (condensed chromatin - CC, karyorrhectic cells - KR, pyknotic cells - PY) and necrosis (karyolitic cells - KL) [16]. All these cell characteristics were analyzed in the samples.

### Statistical analysis

Statistical analysis was performed by descriptive methods (i.e., mean, standard deviation, median, frequency and quartiles); multiple comparisons were accomplished using the analysis of variance (ANOVA) with the *post-hoc* Tukey test. The associations between the studied parameters were evaluated using the Spearman correlation test in the Statistical Package for the Social Sciences (SPSS) software version 20.0. Differences were considered to be significant with p <0.05.

## Results

The general characteristics of patients with respiratory disease and individuals from the control group are provided in Table 1. There was a predominance of male subjects, caucasians and adults in both case and control groups. It is noteworthy that all subjects were in advanced adulthood (>60 years), with the exception of patients with TB. The smoking status was also prevalent among individuals from the case groups, with a high consumption of cigarettes per year.

**Table 1:** Characteristics of research subjects

	COPD	LC	TB	Control
	*n* = 28	*n* = 18	*n* = 22	*n* = 17
Sex (male)	14	13	16	9
Ethnicity (Caucasian)	23	14	10	17
Age (years)^a^	64.21 ± 8.20	65.06 ± 6.64	36.09 ± 16.25	62.82 ± 4.78
BMI^a^	24.47 ± 4.35	24.44 ± 4.13	23.67 ± 3.74	26.40 ± 3.90
Smoking status Never/Ex/Smoker	1/24/3	0/14/4	9/4/9	9/8/0
Cigarretes-year^b,c^	7300 (730–25550)	7300 (1460–21900)	6387 (5475–14600)	7200 (1800–10800)

*n* sample size, *COPD* Chronic Obstructive Pulmonary Disease, *LC* Lung Cancer, *TB* Tuberculosis, *BMI* Body Mass Index. ^a^Expressed as mean ± SD; ^b^Cigarettes smoked per year for smokers and ex-smokers; ^c^ Expressed as median (min.-max.)

The COPD group had a higher frequency of apoptotic cells compared to TB and LC group. The TB group showed a higher frequency of DNA damage, defect in cytokinesis and necrotic cells (Table 2).

**Table 2:** Micronuclei frequencies and cellular abnormalities in COPD, TB, LC patients and control subjects

%	COPD	LC	TB	Control
	*n* = 28	*n* = 18	*n* = 22	*n* = 17
Normal cells	0.15 (0.00–0.30)^a,c^	0.15 (0.05–0.35)^a^	0.65 (0.40–0.90)^d^	0.35 (0.10–3.00)
MN (basal cells)	0.00 (0.00–0.05)	0.00 (0.00–0.05)	0.00 (0.00–0.10)	0.00 (0.00–0.05)
MN (differentiated cells)	0.20 (0.00–0.35)^a,c^	0.15 (0.05–0.30)^a^	0.50 (0.30–0.70)^a,d^	0.30 (0.50–3.00)
Nuclear bud	0.10 (0.00–0.25)^a,c^	0.10 (0.05–0.30)^a^	0.25 (0.10–0.50)^d^	0.20 (0.00–1.20)
Binucleated cells	0.10 (0.05–0.20)^a,c^	0.15 (0.05–0.30)^a^	0.30 (0.10–0.60)^a^	0.65 (0.15–1.55)
Condensed chromatin	0.95 (0.40–2.25)^a,c^	0.97 (0.40–1.90)^a^	2.20 (0.60–6.60)^a,d^	0.05 (0.00–0.60)
Karyorrhectic cells	2.05 (0.75–3.40)^a,b^	1.12 (0.35–1.95)	1.65 (0.80–4.50)^a^	0.25 (0.00–5.00)
Pyknotic cells	3.00 (1.50–3.90)^a,b,c^	1.37 (0.40–3.15)^a^	2.55 (0.80–4.40)^a,d^	0.15 (0.00–0.50)
Karyolitic cells	2.10 (0.60–3.10)^b^	0.85 (0.40–1.95)^a^	2.20 (0.90–3.60)^d^	1.85 (0.40–5.20)

*n* sample size, *COPD* Chronic Obstructive Pulmonary Disease, *LC* Lung Cancer, *TB* Tuberculosis, *MN* Micronucleus. Expressed as median (50 %) and quartiles (25 % – 75 %); ^a^ *p* ≤ 0.05 between cases (COPD or LC or TB) and controls; ^b^ *p* ≤ 0.05 between COPD and LC; ^c^ *p* ≤ 0.05 between COPD and TB; ^d^ *p* ≤ 0.05 between TB and LC

## Discussion

In our study, COPD patients were characterized by a higher frequency of cells with characteristics of programmed cell death and necrosis. In the COPD pathology, the pattern of apoptosis is associated with an increased immune response, especially during exacerbation of the disease [19]. The regulation of apoptosis can be influenced by the inflammatory process that is elevated during exacerbated COPD, thereby contributing to a significant increase in the frequency of apoptosis of circulating lymphocytes compared with stable COPD patients [19]. Cell death by apoptosis is genetically programmed and mediated by caspase activation. In contrast, necrosis can normally be considered as a pathological death caused by physical stress or exposure to highly toxic stimuli [20]. The role of necrosis in the pathogenesis of COPD is poorly understood, but the existence of a form of genetically programmed and regulated necrosis called necroptosis is being investigated [21].

In contrast, patients with TB in this study showed a cellular frequency consistent with problems in cytokinesis, DNA repair and DNA damage. The damage of cells by oxidative stress can lead to MN formation as a result of non-repaired or misrepaired DNA damage or chromosome malsegregation, but these damaged cells may also be susceptible to elimination by apoptosis when the level of damage is very high [8]. A study showed a significant increase in cytogenetic markers (MN) after six months of classical antituberculosis therapy compared to control and untreated subjects [11]. The apoptosis in the cells of patients with tuberculosis is associated with a protective response against infection with *Mycobacterium tuberculosis*, while a necrotic response favors infection by the pathogen [22, 23]. Consistently, *M. tuberculosis* inhibits signaling associated with apoptosis of the host cell, but promotes the induction of programmed necrosis. Dysregulation of the cell death pathways involved in necrosis can promote the release of viable bacilli, thereby leading to the progression of tuberculosis [24]. According to what described above we also observed higher frequency of apoptotic and necrotic cells in TB group. Importantly, the mean age of the TB group was considerably lower than that observed in the other groups (including the control group). This data is very interesting and should be taken into consideration when interpreting the results because it highlights the importance of the disease in the induction of DNA damage even in younger populations.

Studies of human populations exposed to environmental carcinogens have described a positive association between the level of chromosomal lesions and cancer risk [25–27]. LC patients also exhibited higher levels of MN in lymphocytes compared to control patients [26]. Our results differed from those described in the literature because few chromosomal aberrations were observed in LC patients, such as COPD patients. Patients with LC and COPD make use of various drugs, have chronic diseases and use tobacco [28, 29]. COPD patients also use inhaled corticosteroids, which can reduce DNA damage and minimize the formation of MN [30, 31]. Additionaly, continuous exposure to mutagenic and carcinogenic agents moderately can trigger an adaptive response that will protect against future DNA damage [32].

The control subjects in this study showed a frequency of DNA damage and defects in cytokinesis within the predicted normal range for the population (0.5–2.5 MN per 1,000 cells) [12]. Cells showing MN are rarely found in healthy individuals, but become more common in individuals exposed to radiation or other genotoxic agents. It is noteworthy that the control group was composed of individuals with advanced adult age and possible comorbidities, factors that may have contributed to the observed results. The determination of the MN frequency and other cellular abnormalities can be used to quantify permanent DNA damage and the frequency of chromosomal aberrations in patients with COPD, TB or LC. MN frequencies are extensively used in molecular epidemiology and cytogenetic analyses to assess the presence and the extent of chromosomal damage in human populations exposed to genotoxic agents or with a genetic profile susceptible to DNA damage [12, 18]. Monitoring of DNA damage in pathological situations has been investigated because it can add a new dimension to clinical expression and may also represent a potential target for therapeutic intervention [4–6, 8].

## Conclusion

COPD patients showed cellular abnormalities that corresponded to cell death by apoptosis and necrosis, while patients with TB showed defects in cytokinesis and dysfunctions in DNA repair that resulted in the formation of MN besides apoptotic and necrotic cells. Understanding the mechanisms by which this process occurs is essential for the improvement of strategies for the prevention and treatment of lung diseases.
